# Divorce and subsequent increase in uptake of antidepressant medication: a Finnish registry-based study on couple versus individual effects

**DOI:** 10.1186/s12889-015-1508-9

**Published:** 2015-02-19

**Authors:** Christiaan WS Monden, Niina Metsä-Simola, Saska Saarioja, Pekka Martikainen

**Affiliations:** Department of Sociology, Manor Road, OX1 3UQ Oxford, United Kingdom; Population Research Unit, University of Helsinki, Helsinki, Finland

**Keywords:** Divorce, Antidepressants, Mental health, Couples, Marriage, Finland

## Abstract

**Background:**

There is an average negative mental health effect for individuals who experience divorce. Little is known whether the pattern of such divorce effects varies within couples. We study whether the husband and wife experience similar harmful effects of divorce, whether they experience opposite effects, or whether divorce effects are purely individual.

**Methods:**

We use Finnish registry data to compare changes over a period of 5 years in antidepressant use of husbands and wives from 4,558 divorcing couples to 108,637 continuously married pairs aged 40–64, all of whom were healthy at baseline.

**Results:**

In the period three years before and after divorce antidepressant use increases substantially. However, the likelihood of uptake of antidepressant medication during this process of divorce by one partner appears to be independent of medication uptake in the other partner. In contrast, among continuously married couples there is a clear pattern of convergence: If one partner starts to use antidepressants this increases the likelihood of uptake of antidepressant medication in the other partner.

**Conclusions:**

Our findings suggest that divorce effects on antidepressant use are individual and show no pattern of either convergence or divergence at the level of the couple. The increased incidence of antidepressant use associated with divorce occurs in individuals independent of what happens to their ex-partner.

**Electronic supplementary material:**

The online version of this article (doi:10.1186/s12889-015-1508-9) contains supplementary material, which is available to authorized users.

## Background

The correlation between divorce and mental health is well-established [1-7]. Poor mental health is both a determinant and a consequence of divorce; the relative importance of causation and selection remains a subject for study [8-10]. Divorce can be thought of as a health-risk to which both partners in a divorcing couple are exposed. This leads to the following question: Do similar mental health effects of divorce occur in both partners or are divorce effects purely individual? In other words, as both partners are exposed to the divorce, we might find that their health is affected similarly (convergence); they may have opposing reactions with one being better off than the other (divergence) or their reactions may be individual and uncorrelated (independence).

Several studies have taken into account the pre-divorce characteristics of both partners when studying determinants [11] or consequences of divorce [12]. However, we are not aware of studies that compare the health outcomes of the two partners within a couple during and after divorce. We assume the focus on the individual is driven by a lack of data sources that follow both partners after divorce [13]. In longitudinal household surveys, such as the British Household Panel Study for instance, often only one of the partners remains in the study after divorce. Couples where both ex-partners remain in the study may be rather selective (i.e. low conflict divorce or no mental health effects). Register data overcome such selective attrition problems and allow us to study if and how divorce affects both partners.

We analyse Finnish registry data to compare changes in use of antidepressant medication for both partners in divorcing couples and a comparison group of continuously married couples. Our analysis is guided by the idea that there are three possible patterns of health change in divorcing couples – convergence, divergence and independence – and that it is an empirical question which pattern is actually observed. Below we briefly elaborate on each pattern.

### Convergence

Although partners may have different experiences of the divorce process, they will share a substantial number of stressors. Especially around the peak of the divorce process we might expect partners to be exposed to similar stressors, such as fights and conflicts between the partners. Other examples are tensions caused by loyalty issues and loss of contacts with regard to mutual friends and family members, issues about care arrangements for the children, and questions about housing arrangements and dividing joint assets. The number of shared stressors will be smaller the further we move away from the peak in the divorce process. Both partners experience a loss in economies of scale and companionship (the exception being when one of the partners immediately moves in with a new partner). If the effects of a divorce are mainly driven by characteristics at the couple level, we would expect that both partners are affected in rather similar ways. In couples with many stressors and escalation of the divorce process, both partners would be affected strongly negatively, whereas in couples with a less stressful process, neither partner would be affected or both would only experience small effects. In sum, if divorce affects both partners roughly similarly, we expect a pattern of convergence, in particular in the short term.

Continuously married couples experience shared environments and it seems reasonable to expect convergence between married partners. There is quite some evidence for convergence in marriage for several mental health conditions and depressive symptoms in particular. For general health indicators, there is little evidence for convergence [14], but cross-sectional clinical studies of couples where one partner is a patient have reported concordance for major depression [15,16] and depressive symptoms [17,18], although not all studies found this pattern [19]. Also population-based samples have reported spousal concordance for major depression and related medication [20-22]. Shared experiences lead to the same outcomes, but it can also be that a health change in one partner leads to a change in the other partner. The two mechanisms are difficult to separate and both may be operating simultaneously. If the impact of divorce is particularly strong, convergence in divorcing couples should be at least as strong in divorcing couples as in married couples.

### Divergence

The competing hypothesis of divergence emphasizes that divorce often is a case of asymmetry. This hypothesis argues that spouses have different motives for divorce and may have a different experience of the divorce process. It could be that one partner has more to gain or lose from the divorce than the other partner. This asymmetry may be reflected in the health consequences. If the majority of divorces are characterised by asymmetry (or if only in asymmetric cases there are serious health consequences) then we would expect to observe that only one of the two partners develops a mental health condition. Taken to its extreme, if asymmetry characterises all divorce this argument would predict all divorces to result in a situation where either only the ex-husband or only the ex-wife experiences a (negative) heath effect. Particularly in cases where one of the spouses finds a new partner and the other does not, we would expect to find a pattern of divergence. Divergence may also be more likely when dependent children are involved (as they tend to live with one of the ex-partners most of the time).

### Independence

Finally, we would expect to find no correlation between the health changes of the partners if the effects of divorce are purely individual. It could be that partners are exposed to the same health hazard – the divorce – but their reaction is idiosyncratic. Alternatively, it could be that the partners experience the divorce process itself in different, but not opposite, ways. This means that they are, in fact, not exposed to the same health risk and hence their outcomes will differ. The literature on the gendered nature of divorce supports this idea by showing that the determinants of divorce are not necessarily the same for the two partners in a marriage [23]. Especially in the longer run, when both partners have gone their own ways and experience different social environments, a pattern of purely individual effects might become increasingly likely.

## Methods

### Registry linkage and sampling

This study uses population registration data on Finnish couples living together with both spouses aged 40 to 64. The lower age limit is data driven; we set the upper age limit to retain a more homogenous group with regard to retirement and pensions and to avoid issues with selective mortality. The data are derived from a two-stage random sample. First, a simple random sample covering 14% of individuals aged 40 or over and living in private households at the end of 1997 was drawn from population registers. Second, all household members of the selected individuals and information on their family relationships were added to the sample. The data include socio-demographic information at the end of 1997 and dates of divorces from 1.1.1998 to 31.12.2003. These data were further linked to information on mortality and purchases of prescription medication from 1.1.1995 to 31.12. 2007. The information on purchases on prescription medication is provided by the Social Insurance Institution and other information by Statistics Finland, which also performed the data linkage using personal identification codes.

### Ethics

The ethics committee of Statistics Finland has approved the study (permission TK-53-574-04) and the data are available to researchers in an anonymous format.

### Measurements

Information on all medications prescribed by a physician and bought from any pharmacy in the country is forwarded to Social Insurance Institution as part of the national drug reimbursement scheme. In Finland, antidepressants are sold to the public only by authorised pharmacies against a prescription issued by a medical doctor, and no over-the-counter antidepressants are available. All residents are eligible for reimbursement as part of the public health insurance. Drug costs are directly reimbursed at the time of purchase and the reimbursement covers a maximum prescription of three months at a time. The national Prescription Register, which is updated monthly from all retail pharmacies, contains individual-level information on all reimbursed medications. The register includes information about the day of purchase and the type of medication classified according to the WHO Anatomical Therapeutic Chemical (ATC). We extracted all information on purchases of antidepressants (ATC-codes N06A) and use uptake of antidepressant medication as an indicator of mental health. Indicators for milder distress symptoms were not available in the data that we had access to.

We used two measures of socioeconomic position: education and household income. The three educational categories were based on the highest completed degree or certificate: tertiary education, intermediate education, and basic education or less or unknown (the latter concerns the few cases where the highest education may be unknown if educational certificates were obtained abroad and not reported later). Household disposable income per consumption unit was used to measure income. All taxable income sources for all household members were included: wages, capital income and taxable income transfers, but excluding taxes. In the calculation of household consumption units the first household member was weighted as 1.0 unit, second adult as 0.7 units and children as 0.5 units. This corresponds to the OECD equivalence scale [24]. Income was divided into quintiles with cut-off points calculated at the household level. We further measured the number of dependent children aged 18 or less living in the household, and separated between the following categories; none, one, two, and three or more.

### Analytic sample

We restricted the sample to households with two adults aged 40 or over and married to each other. Couples in which either spouse died or emigrated during the period from the beginning of 1998 to the end of 200 were excluded. We further restricted the sample to couples that were healthy at baseline (see definition of baseline below); i.e. spouses had not used antidepressants in the third year before divorce (year 1998 for the continuously married), thus excluding 9.5% of the sample. Couples where one or both of the partners used antidepressant at baseline had a higher divorce rate than those where neither of the partners was on medication (see Table 1). Excluding couples with prior medication use simplifies our analysis because we can model new medication use during and after the divorce process. A change from medication before divorce to no medication after the divorce is more difficult to interpret. Excluding couples with prior medication left us with an analytic sample of 113,195 couples of whom 4,558 (4%) were divorced by 2003. Table 2 presents descriptive statistics for the variables in the analyses.

**Table 1:** **Husband’s and wife’s antidepressant medication use at baseline by marital trajectory**

**Husband**	**Wife**	**Continuously married N %**		**Divorcing N %**	
None	None	108,637	90.8	4,558	82.8
None	Medication	6,644	5.6	530	9.6
Medication	None	3,699	3.1	324	5.9
Medication	Medication	626	0.5	90	1.6
*Total*		119,606	100	5,502	100

**Table 2:** **Socio-demographic composition of couples by marital trajectory**

**Husband’s age**	**N**	**%**	**N**	**%**
40-44	15,487	14.3	1,357	29.8
45-49	27,999	25.8	1,659	36.4
50-54	27,867	25.7	980	21.5
55-59	21,003	19.3	401	8.8
60-64	16,281	15.0	161	3.5
**Wife’s age**				
40-44	25,116	23.1	2,038	44.7
45-49	29,700	27.3	1,448	31.8
50-54	26,574	24.5	749	16.4
55-59	18,088	16.6	243	5.3
60-64	9,159	8.4	80	1.8
**Husband’s education**				
Basic or unknown	41,928	38.6	1,425	31.3
Intermediate	33,422	30.8	1,643	36.0
Tertiary	33,287	30.6	1,490	32.7
**Wife’s education**				
Basic or unknown	39,981	36.8	1,261	27.7
Intermediate	37,892	34.9	1,721	37.8
Tertiary	30,764	28.3	1,576	34.6
**Household net income quintiles**				
Lowest	21,188	19.5	1,130	24.8
2.	21,430	19.7	925	20.3
3.	21,818	20.1	889	19.5
4.	22,127	20.4	808	17.7
Highest	22,074	20.3	806	17.7
**Children under 18**				
No	66,183	60.9	1,767	38.8
Yes	42,454	39.1	2,791	61.2

### Follow-up periods

An important issue in longitudinal studies of divorce is the timing of the baseline measurement and follow-ups vis-a-vis the time of divorce. It is convenient to treat divorce as an event happening at a specific moment in time – often the official administrative date of the divorce is used – but, of course, divorce is a process. Months or even years before the official date, the partners may have concluded that the marriage was not working anymore and have decided to divorce. This process prior to divorce date is stressful and health changes may already be observed over this period. Baseline observations close to the official divorce date will pick-up some of the negative effects that the divorce process may have on health (i.e. the crisis effect). Furthermore, it matters whether one wants to learn more about the immediate effects of the divorce process during the crisis period, or whether the focus is on short-term effects in the first few years after the divorce when, for instance, remarriage becomes important. In this study, we investigate health changes during the process (crisis period) and changes between the baseline and two years after the divorce (short-term consequences). Long-term effects are beyond the scope of this study.

**Figure 1:**
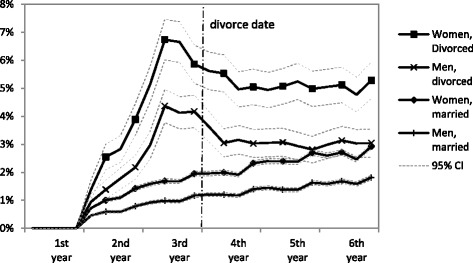
**Prevalence of antidepressant use by sex and martial trajectory.**

Divorced individuals were followed for antidepressant use from the third year before divorce (baseline) to the second year after divorce, and the continuously married were observed from 1998 to 2003. In Figure 1, we plot antidepressant medication purchases in three month intervals for divorced and continuously married men and women. The highest prevalence of antidepressant use is observed about 6 to 9 months before the actual divorce; roughly concurrent with the timing of the initiation of formal divorce proceedings. After that, antidepressant use declines for the next 12 months. For continuously married individuals there is a moderately increasing trend in medication use throughout the follow-up period. For both groups we compare the baseline to a three year follow-up and a five year follow-up. For married couples, the baseline is always 1998 with the first follow-up referring to 2001 and the second referring to 2003. We label these two time–frames as “crisis period” and “short-term consequences” for divorcing couples. Thus, in the “crisis-period” we examine uptake of antidepressant medication between the baseline (third year before divorce) and the 12 months before the divorce date. For “short-term consequences” we examine uptake between the baseline and the second year after divorce.

### Statistical methods

We use logistic regression to estimate the association between partners’ incidence of new antidepressant medication use. Separate models for the two follow-up periods are estimated. We regress wife’s antidepressant medication use on husband’s antidepressant use, and vice versa. Subsequently, we estimate the effects of divorce status and the interaction term between divorce status and spouse’s antidepressant use. From these analyses we can determine (1) whether there is an overall divorce-effect on incidence of antidepressant medication use and (2) whether the association between the partners’ medication differs between married couples and divorcing couples. We control for the effects of age and education of both spouses’, household income and resident underage children. To account for the possible increasing trend in antidepressant utilisation patterns, we also controlled for time of medication purchase, but the effect was negligible. All analyses were conducted in Stata 11.

## Results

### Descriptive results

Table 3 provides a cross-tabulation of husband’s and wife’s change in medication by marital trajectory. At the individual level, there is a clear negative association between medication uptake and divorce, especially in the crisis period. Among women, 3.7% (3.5% + 0.2%) of the continuously married individuals start using antidepressants between baseline and two years later compared to 12.2% among women who divorce, whereas the figures form men are considerable lower with 2.2% and 8.4% for continuously married and divorcing men respectively. This is in line with previous findings on other health outcomes [25,26].

**Table 3:** **Antidepressant medication use of husband and wife by marital trajectory and follow-up period**

			**First follow-up (crisis period)**		**Second follow-up (short-term consequences)**	
			**Husband**		**Husband**	
			No	Yes	No	Yes
Continuously married	Wife	No	102,507	2,159	101,082	2,610
			94.4%	2.0%	93.0%	2.4%
		Yes	3,785	186	4,568	377
			3.5%	0.2%	4.2%	0.3%
Divorcing	Wife	No	3,671	332	3,915	222
			80.5%	7.3%	85.9%	4.9%
		Yes	505	50	390	31
			11.1%	1.1%	8.6%	0.7%

Also at the couple level we observe a clear negative association between divorce and uptake of antidepressant medication. For the crisis-period, in only one in eighteen continuously married couples (5.6%) do we observe uptake by either husband (2%), wife (3.5%) or both partners (0.2%). Among divorced couples, almost one in five couples (19.5%) experienced an incidence of antidepressant use in one or both partners.

It is rare for both partners in a married couple to start medication; we only observe it in 186 couples or 0.2% of all married couples in the first follow-up period. In the second period this number has increased to 377, still only 0.3%. Among the divorcing couples, joint antidepressant incidence is rare too in absolute terms (1.1% in the first follow-up period), but it is more likely compared to continuously married couples. Note that these figures do not take into account socio-demographic differences between the two types of couples. Especially the age difference between continuously married and divorcing couples is substantial and this is important as age is related to the use of antidepressant medication. We therefore move on to multivariate analysis and test the competing hypotheses explicitly.

### Logistic model

In Table 4, we present the partners’ similarity in health change in Odds Ratio’s (OR) while taking into account age, education, income, and number of dependent children under 18 among those healthy at baseline. The OR describes the association between the husband’s and wife’s chance of medication uptake. An OR of 1 indicates purely individual effects, an OR lower than 1 is in line with divergence and an OR higher than 1 reflects convergence.

**Table 4:** **Odds ratios for uptake of antidepressant medication in men and women by marital trajectory (panel A) and by spouse’s uptake of antidepressant medication (panel B)**

	**Men**				**Women**			
	**First follow-up (crisis period)**		**Second follow-up (short term consequences)**		**First follow-up (crisis period)**		**Second follow-up (short term consequences)**	
Panel A								
Marital trajectory	OR	95% CI	OR	95% CI	OR	95% CI	OR	95% CI
Continuously married couples	1.00	ref	1.00	ref	1.00	ref	1.00	ref
Divorcing couples	4.05	3.61 – 4.54	1.99	1.74 – 2.28	3.68	3.35 – 4.06	2.06	1.86 – 2.30
Panel B								
Spouse’s antidepressant use	OR	95% CI	OR	95% CI	OR	95% CI	OR	95% CI
Continuously married couples								
No	1.00	ref	1.00	ref	1.00	ref	1.00	ref
Yes	2.33	2.00 – 2.72	3.19	2.85 – 3.57	2.33	2.00 – 2.72	3.19	2.85 – 3.57
Divorcing couples								
No	1.00	ref	1.00	ref	1.00	ref	1.00	ref
Yes	1.10	0.80 – 1.50	1.39	0.94 – 2.05	1.10	0.80 – 1.50	1.39	0.94 – 2.05

Note. p < 0.001 for interaction term of spouse’s uptake of antidepressants and marital trajectory in all models. Adjusted for spouses’ age, education, household income deciles and presence of children in the household.

The results in the first row in Table 4 first of all confirm that divorce is associated with a higher likelihood of medication uptake. In the crisis-period (i.e. first follow-up), men in divorcing couples are four times more likely (OR = 4.05) to start using antidepressant medication compared to their counterparts in continuously married couples. For women the odds of uptake are about 3.68 times higher among those in divorcing couples.

The multivariate model also confirms the husband-wife patterns described in Table 3. We find an OR of 2.33 (95% CI = 2.00-2.72) for men in married couples in the first follow-up period, indicating a tendency for men to start antidepressant use if their spouse is also starting to take antidepressant medication during this period. (Note that simultaneous incidence means that both partners use medication at some point during a 1-year period, making overlap highly likely but not strictly necessary.) The OR among divorcees is much lower at 1.10 (95% CI = 0.80-1.50) and, moreover, is not significantly different from 1. Although we observe more health changes among individuals in the divorcing couples, these changes appear to be mainly individual. The interaction term between spouse’s medication uptake and couple type confirms that the odds ratio’s for continuously married and divorcing couples are significantly different.

The results for short-term consequences (i.e. the second follow-up period) show a largely similar pattern. The association between medication uptake and divorce is less strong but again we observe a pattern of convergence in continuously married couples. The clustering over a longer period of time appears stronger; the OR increases from 2.33 to 3.19 among married men. And again the pattern for divorcing couples is significantly different from the continuously married. The odds ratio for divorcing couples is 1.39 (95% CI = 0.94-2.05); not significantly different from 1.

Finally, we checked whether the pattern depends on repartnering or the presence of children prior to divorce. Splitting the sample into couples with resident children (48% of the divorcing couples) and couples without resident children at baseline resulted in highly similar findings for the two groups (see Additional file 1: Table S1). With regard to repartnering the divorcing couples fall into four groups. In 2,576 out of 4,558 divorcing couples (56.5%), neither of the partners is living with a new partner two years after the divorce, whereas in one in 10 cases (448 couples) both divorcees are living with a new partner. In the remaining cases, only one of the partners is living with a new partner and it is more common that this is the male partner than the female partner (21.4% and 12.2% of all divorcing couples respectively). Splitting the results by repartnering showed no systematic pattern across the four groups (see Additional file 2: Table S2).

## Discussion

We examined patterns in anti-depressant uptake in couples in the period just before and shortly after divorce using Finnish registry data on couples aged 40 and over. In divorcing couples, the uptake of anti-depressants is significantly and substantially higher than in couples who stay married throughout the observation period. The increased risk of using anti-depressant medication, however, seems to be uncorrelated between partners in divorcing couples. With regard to married couples our results confirm known findings of convergence of spousal health in the literature. Before drawing conclusions and elaborating on their implications, we need to address some limitations and strengths of our study.

We checked the robustness of the results for two important issues. First, our analytical sample is limited to couples where both partners do not use antidepressants at baseline. However, similar results to those reported here are found for the full sample including couples with one or two unhealthy partners at baseline. Second, there might have been exogenous changes in medication use in the observation period. There indeed seems to be an increasing trend in medication use but adding calendar year to the models to take this into account did not change the results reported here.

Furthermore, our findings are limited to antidepressant use and any conclusions should not be generalized to overall health or well-being. It should also be noted that (tricyclic) anti-depressants are sometimes prescribed for chronic pain management rather than mental health conditions (for antidepressant utilisation in Finland see for instance Sihvo et al. [27]). It might well be that there are stronger couple effects for other types of health outcomes and/or less severe health changes. Research on divorce has often showed stronger associations of divorce with overall satisfaction, general health or depressive symptoms than with more severe and clinical conditions. We encourage replication of our couple design with different and milder health outcomes.

The association between health and divorce has been found throughout Western societies and one would expect similar patterns to those reported here for other countries as well. Replication of our study would show to what extent this holds true. Countries differ in divorce rates, antidepressant use and how individualistic they are (in norms and values as well as, for instance, taxation and welfare). These differences might affect the association between divorce and health and the degree to which associations are formed at the individual or couple level.

We also have to note that our data do not include young divorcees (below age 40) by design. Again, the associations in a younger group might differ from those found here. The use of antidepressants is lower in that age group and the implications of divorce could be different, for instance because there might be more couples without children and where there are children they will be younger than in the current data. We look forward to replications of our approach with other outcomes, in younger age groups and in other countries.

## Conclusions

Based on antidepressant uptake we found no support for strong couple-level effects of divorce. Although there is clearly an increased risk of antidepressant uptake at the time of divorce for individuals, there is no convergence of this risk within divorcing couples. We did not find evidence for the opposite hypothesis of divergence either: There is no overrepresentation of winner/loser type couples where only one of the partners starts to take antidepressant medication after divorce. Rather, we conclude that the increased incidence of antidepressant use associated with divorce occurs in individuals independent of what happens to their ex-partner. In contrast, among the married there is strong evidence for the convergence of antidepressant use.

What are the implications of these findings for our understanding of divorce effects? The results suggest that the divorce process is different not only from couple to couple, but also for the individuals within one couple. It thus seems likely that the partners experience the divorce process itself in ways that are completely unrelated. This can be interpreted to mean that they are not exposed to the same health risk at the time of divorce and their mental health outcomes thus differ. Furthermore, with increasing time after divorce partners also experience increasingly different social environments and purely individual effects become increasingly likely. However, this does not rule out that under certain conditions (i.e. in particular sub-sets of couples) patterns of convergence or divergence occur. At the individual level divorce effects are heterogeneous [28] and various modifiers have been identified, such as marital quality [29] or the presence and ages of children [30]. In these Finnish data, re-partnering and presence of children do not appear to be such modifiers for couple level effects on mental health, but many other possible modifiers both at the individual and couple level remain to be examined. However, overall the results imply that although marriage counselling is best carried out at the couple level, post-divorce policies to support divorcees need to target individuals.

## Additional files

Additional file 1:
**Odds rations for uptake of antidepressant medication in men and women by marital trajectory (panel A) and by spouse’s uptake of antidepressant (panel B) by presence of children in the household.**
Additional file 2:
**Odds ratios for uptake of antidepressant medication at second follow-up by sex and partner status.**
