# Supplementary material for: Divorce and subsequent increase in uptake of antidepressant medication: a Finnish registry-based study on couple versus individual effects
Source: BMC Public Health. 2015 Feb 19;15:158. doi: 10.1186/s12889-015-1508-9 (PMC4341230; doi:10.1186/s12889-015-1508-9)
Supplement: Additional file 1: — Odds rations for uptake of antidepressant medication in men and women by marital trajectory (panel A) and by spouse’s uptake of antidepressant (panel B) by presence of children in the household. [file 12889_2015_1508_MOESM1_ESM.docx]

| **Table S1. Odds ratios for uptake of antidepressant medication in men and women by marital trajectory (panel A) and by spouse’s uptake of antidepressant medication (panel B) by presence of children in the household.** | | | | | | | | | | | | | | | | | | | | |
| --- | --- | --- | --- | --- | --- | --- | --- | --- | --- | --- | --- | --- | --- | --- | --- | --- | --- | --- | --- | --- |
|  |  | Men | | | | | | | | |  | Women | | | | | | | | |
| Children present | | First follow-up (crisis period) | | | |  | Second follow-up (short term ) | | | |  | First follow-up (crisis period) | | | |  | Second follow-up (short term) | | | |
| Panel A | | OR | 95% CI | | |  | OR | 95% CI | | |  | OR | 95% CI | | |  | OR | 95% CI | | |
| Marital trajectory | |  |  |  |  |  |  |  |  |  |  |  |  |  |  |  |  |  |  |  |
|  | Continuously married | 1.00 |  | ref |  |  | 1.00 |  | ref |  |  | 1.00 |  | ref |  |  | 1.00 | ref |  |  |
|  | Divorcing | 4.52 | 3.87 | - | 5.28 |  | 2.03 | 1.68 | - | 2.44 |  | 3.43 | 2.98 | - | 3.95 |  | 2.16 | 1.86 | - | 2.50 |
|  |  |  |  |  |  |  |  |  |  |  |  |  |  |  |  |  |  |  |  |  |
| Panel B | | OR | 95% CI | | |  | OR | 95% CI | | |  | OR | 95% CI | | |  | OR | 95% CI | | |
| Spouse's antidepressant use | |  |  |  |  |  |  |  |  |  |  |  |  |  |  |  |  |  |  |  |
|  | Continuously married couples |  |  |  |  |  |  |  |  |  |  |  |  |  |  |  |  |  |  |  |
|  | No | 1.00 |  | ref |  |  | 1.00 |  | ref |  |  | 1.00 |  | ref |  |  | 1.00 | ref |  |  |
|  | Yes | 2.34 | 2.00 | - | 2.72 |  | 3.19 | 2.85 | - | 3.57 |  | 2.34 | 2.01 | - | 2.72 |  | 3.19 | 2.86 | - | 3.57 |
|  | Divorcing couples |  |  |  |  |  |  |  |  |  |  |  |  |  |  |  |  |  |  |  |
|  | No | 1.00 |  | ref |  |  | 1.00 |  | ref |  |  | 1.00 |  | ref |  |  | 1.00 | ref |  |  |
|  | Yes | 1.03 | 0.66 | - | 1.60 |  | 1.68 | 1.01 | - | 2.80 |  | 1.03 | 0.66 | - | 1.61 |  | 1.69 | 1.02 | - | 2.82 |
|  |  |  |  |  |  |  |  |  |  |  |  |  |  |  |  |  |  |  |  |  |
| No children present | |  |  |  |  |  |  |  |  |  |  |  |  |  |  |  |  |  |  |  |
| Panel A | | OR | 95% CI | | |  | OR | 95% CI | | |  | OR | 95% CI | | |  | OR | 95% CI | | |
| Marital trajectory | |  |  |  |  |  |  |  |  |  |  |  |  |  |  |  |  |  |  |  |
|  | Continuously married | 1.00 |  | ref |  |  | 1.00 |  | ref |  |  | 1.00 |  | ref |  |  | 1.00 | ref |  |  |
|  | Divorcing | 3.63 | 3.09 | - | 4.26 |  | 1.96 | 1.63 | - | 2.35 |  | 3.87 | 3.41 | - | 4.38 |  | 1.97 | 1.70 | - | 2.28 |
|  |  |  |  |  |  |  |  |  |  |  |  |  |  |  |  |  |  |  |  |  |
| Panel B | | OR | 95% CI | | |  | OR | 95% CI | | |  | OR | 95% CI | | |  | OR | 95% CI | | |
| Spouse's antidepressant use | |  |  |  |  |  |  |  |  |  |  |  |  |  |  |  |  |  |  |  |
|  | Continuously married couples |  |  |  |  |  |  |  |  |  |  |  |  |  |  |  |  |  |  |  |
|  | No | 1.00 |  | ref |  |  | 1.00 |  | ref |  |  | 1.00 |  | ref |  |  | 1.00 | ref |  |  |
|  | Yes | 2.34 | 2.01 | - | 2.72 |  | 3.19 | 2.85 | - | 3.57 |  | 2.34 | 2.00 | - | 2.72 |  | 3.19 | 2.85 | - | 3.57 |
|  | Divorcing couples |  |  |  |  |  |  |  |  |  |  |  |  |  |  |  |  |  |  |  |
|  | No | 1.00 |  | ref |  |  | 1.00 |  | ref |  |  | 1.00 |  | ref |  |  | 1.00 | ref |  |  |
|  | Yes | 1.18 | 0.76 | - | 1.83 |  | 1.09 | 0.59 | - | 2.00 |  | 1.19 | 0.77 | - | 1.84 |  | 1.08 | 0.59 | - | 2.00 |
| *Note.* p<0.001 for interaction term of spouse’s uptake of antidepressants and marital trajectory in all models. | | | | | | | | | | | | | | | | |  |  |  |  |
| Adjusted for spouses' age, education, and household income deciles. | | | | | | | |  |  |  |  |  |  |  |  |  |  |  |  |  |
|  |  |  |  |  |  |  |  |  |  |  |  |  |  |  |  |  |  |  |  |  |
