# Supplementary material for: Divorce and subsequent increase in uptake of antidepressant medication: a Finnish registry-based study on couple versus individual effects
Source: BMC Public Health. 2015 Feb 19;15:158. doi: 10.1186/s12889-015-1508-9 (PMC4341230; doi:10.1186/s12889-015-1508-9)
Supplement: Additional file 2: — Odds ratios for uptake of antidepressant medication at second follow-up by sex and partner status. [file 12889_2015_1508_MOESM2_ESM.docx]

| **Table S2.** | |  |  |  |  |  |  |  |  |  |  |  |  |  |  |  |  |  |  |  |
| --- | --- | --- | --- | --- | --- | --- | --- | --- | --- | --- | --- | --- | --- | --- | --- | --- | --- | --- | --- | --- |
| **Odds ratios for uptake of antidepressant medication at second follow-up by sex and partner status.** | | | | | | | | | | | | | | | | | | | | |
|  |  | Husband and wife both single | | | | | | | | |  | Husband and wife both repartnered | | | | | | | | |
|  |  | men | | | |  | women | | | |  | men | | | |  | women | | | |
| Panel A | | OR | 95% CI | | |  | OR | 95% CI | | |  | OR | 95% CI | | |  | OR | 95% CI | | |
| Marital trajectory | |  |  |  |  |  |  |  |  |  |  |  |  |  |  |  |  |  |  |  |
|  | Continuously married | 1.00 |  | ref |  |  | 1.00 |  | ref |  |  | 1.00 |  | ref |  |  | 1.00 | ref | | |
|  | Divorcing | 2.52 | 2.15 | - | 2.95 |  | 2.13 | 1.86 | - | 2.44 |  | 1.68 | 1.08 | - | 2.60 |  | 1.81 | 1.29 | - | 2.54 |
|  |  |  |  |  |  |  |  |  |  |  |  |  |  |  |  |  |  |  |  |  |
| Panel B | | OR | 95% CI | | |  | OR | 95% CI | | |  | OR | 95% CI | | |  | OR | 95% CI | | |
| Spouse's antidepressant use | |  |  |  |  |  |  |  |  |  |  |  |  |  |  |  |  |  |  |  |
|  | Divorcing couples |  |  |  |  |  |  |  |  |  |  |  |  |  |  |  |  |  |  |  |
|  | No | 1.00 |  | ref |  |  | 1.00 |  | ref |  |  | 1.00 |  | ref |  |  | 1.00 | ref | | |
|  | Yes | 1.22 | 0.75 | - | 1.98 |  | 1.22 | 0.75 | - | 1.97 |  | 1.90 | 0.53 | - | 6.79 |  | 1.88 | 0.53 | - | 6.72 |
|  |  |  |  |  |  |  |  |  |  |  |  |  |  |  |  |  |  |  |  |  |
|  | | Wife repartnered, husband single | | | | | | | | |  | Husband repartnered, wife single | | | | | | | | |
|  |  | men | | | |  | women | | | |  | men | | | |  | women | | | |
| Panel A | | OR | 95% CI | | |  | OR | 95% CI | | |  | OR | 95% CI | | |  | OR | 95% CI | | |
| Marital trajectory | |  |  |  |  |  |  |  |  |  |  |  |  |  |  |  |  |  |  |  |
|  | Continuously married | 1.00 |  | ref |  |  | 1.00 |  | ref |  |  | 1.00 |  | ref |  |  | 1.00 | ref | | |
|  | Divorcing | 1.74 | 1.18 | - | 2.57 |  | 1.84 | 1.36 | - | 2.48 |  | 0.96 | 0.65 | - | 1.41 |  | 2.12 | 1.71 | - | 2.64 |
|  |  |  |  |  |  |  |  |  |  |  |  |  |  |  |  |  |  |  |  |  |
| Panel B | | OR | 95% CI | | |  | OR | 95% CI | | |  | OR | 95% CI | | |  | OR | 95% CI | | |
| Spouse's antidepressant use | |  |  |  |  |  |  |  |  |  |  |  |  |  |  |  |  |  |  |  |
|  | Divorcing couples |  |  |  |  |  |  |  |  |  |  |  |  |  |  |  |  |  |  |  |
|  | No | 1.00 |  | ref |  |  | 1.00 |  | ref |  |  | 1.00 |  | ref |  |  | 1.00 | ref | | |
|  | Yes | 0.86 | 0.20 | - | 3.74 |  | 0.86 | 0.20 | - | 3.74 |  | 2.82 | 1.11 | - | 7.19 |  | 2.86 | 1.13 | - | 7.29 |
| *Note.* p<0.001 for interaction term of spouse’s uptake of antidepressants and marital trajectory in all models. | | | | | | | | | | | | | | | | |  |  |  |  |
| Adjusted for spouses' age, education, household income deciles and presence of children in the household. | | | | | | | | | | | | | | | |  |  |  |  |  |
